# A combined computational and structural model of the full-length human prolactin receptor

**DOI:** 10.1038/ncomms11578

**Published:** 2016-05-13

**Authors:** Katrine Bugge, Elena Papaleo, Gitte W. Haxholm, Jonathan T. S. Hopper, Carol V. Robinson, Johan G. Olsen, Kresten Lindorff-Larsen, Birthe B. Kragelund

**Affiliations:** 1 Structural Biology and NMR Laboratory, Department of Biology, University of Copenhagen, Ole Maaløes Vej 5, DK-2200 Copenhagen N, Denmark; 2 Physical and Theoretical Chemistry Laboratory, Department of Chemistry, University of Oxford, South Parks Road, Oxford OX1 3QZ, UK

## Abstract

The prolactin receptor is an archetype member of the class I cytokine receptor family, comprising receptors with fundamental functions in biology as well as key drug targets. Structurally, each of these receptors represent an intriguing diversity, providing an exceptionally challenging target for structural biology. Here, we access the molecular architecture of the monomeric human prolactin receptor by combining experimental and computational efforts. We solve the NMR structure of its transmembrane domain in micelles and collect structural data on overlapping fragments of the receptor with small-angle X-ray scattering, native mass spectrometry and NMR spectroscopy. Along with previously published data, these are integrated by molecular modelling to generate a full receptor structure. The result provides the first full view of a class I cytokine receptor, exemplifying the architecture of more than 40 different receptor chains, and reveals that the extracellular domain is merely the tip of a molecular iceberg.

## Abstract (web summary)

The prolactin receptor consists of a folded extracellular domain, a transmembrane domain and an intracellular intrinsically disordered domain. Here the authors use a combined experimental and computational approach to obtain a structure of a class I cytokine receptor, the human prolactin receptor.

## Graphical abstract

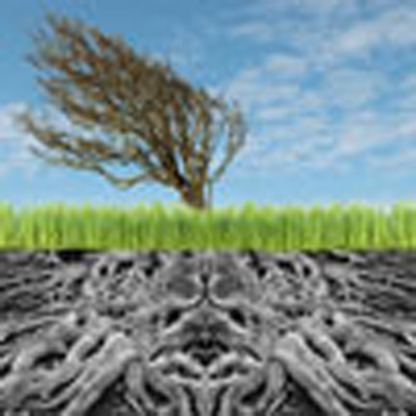

The prolactin receptor (PRLR) and its primary ligand prolactin (PRL) constitute a complex receptor system, linked to more than 300 biological functions ranging from reproduction and cell differentiation to immune responses^1^,^2^. It is best known for its role in mammary gland development and lactation^3^ as well as the pathology hyperprolactinemia^4^, but has also been linked to reproductive disorders^5^ as well as breast^6^,^7^ and prostate^8^ tumorigenesis, and has therefore attracted significant pharmaceutical interest.

The PRLR belongs to the hematopoietic cytokine receptor superfamily, which consists of more than 40 members^9^. It is considered an archetype of the homodimeric group 1 of the family^9^, constituting the simplest cytokine receptors and including, for example, the growth hormone receptor (GHR), the erythropoietin receptor (EPOR) and the thrombopoietin receptor^9^. All these receptors lack intrinsic kinase activity, making them dependent on associated kinases such as the Janus kinases (JAKs) to mediate signalling^9^. They are single-pass transmembrane (TM) proteins with similar overall topologies: (1) a folded extracellular domain (ECD) responsible for ligand binding; (2) a TM domain (TMD) connecting the extracellular- and intracellular parts and (3) an intrinsically disordered intracellular domain (ICD) orchestrating downstream signalling^9^,^10^. Their ECDs fold into two fibronectin type III domains, named D1 (membrane-distal) and D2 (membrane-proximal). The latter contains a conserved WS-motif^11^ that for the PRLR acts as a molecular switch during activation^12^. The ICDs have low-sequence conservation, except for two regions named Box1 and Box2. Box1 is a membrane-proximal proline-rich motif responsible for constitutive association of JAKs^13^, while the function of Box2 remains unclear, although some studies have suggested it to be involved in JAK2 association^13^,^14^,^15^. Recently, studies have revealed that homodimerization of group I cytokine receptors may occur in the absence of hormone, and is insufficient for receptor activation^16^,^17^,^18^. Binding of a hormone to the ECDs leads to the formation of an asymmetric ternary complex consisting of one hormone and two receptor chains^19^,^20^,^21^.

Parts of the PRL/PRLR receptor system have been structurally characterized including structures of the PRL^22^, the 1:1 and 1:2 complexes of PRL:PRLR-ECD^16^,^17^,^18^ and the unliganded human (h) PRLR-ECD-D2 (ref. 12), as well as a recent characterization of the hPRLR-ICD as being intrinsically disordered throughout its length^10^. However, despite substantial efforts, important structural and functional aspects of the PRLR remain uncharacterized. As no structure is available of neither the unliganded PRLR-ECD nor of the TMD, the overall structure and mechanism of signal transfer remains elusive. Signal transduction across the membrane following hormone binding is suggested to occur by subunit rearrangements or propagation of local structural changes, providing the TMDs with a key role. Furthermore, it has been shown for several class 1 cytokine receptors that the ligand-independent homodimerization takes place in the TMDs^16^,^17^,^18^. Despite these significant roles, the TMDs remain largely understudied, although cellular reports on their characteristics and roles in signal transduction are now emerging. A recent study of the GHR proposed a model for signal transduction where the homodimer TMD conformation switches upon receptor activation from a parallel to a left-hand crossover structure, thereby separating the TMDs at the C-terminal end and eventually bringing the two JAK2s into productive positions^17^. However, analogous alanine-insertion studies of the GHR^23^ and the PRLR^24^ suggested that the activation mechanism of the two related receptors differs in a poorly understood manner.

Although high-resolution structures of multi-pass transmembrane proteins in connection with extra-membranous globular domains have been solved (for example, refs 25, 26), no such structures are available of single-pass transmembrane proteins, which have fewer stabilizing contacts in their membrane-embedded region. The structural characterization of the full PRLR is further hampered by the challenges associated with studies of a protein consisting of three structurally diverse domains: a soluble, folded domain, a membrane-embedded domain and an intrinsically disordered domain. Currently, no single high-resolution method is capable of describing the structural characteristics of a protein of this size and complexity. To overcome these inherent limitations in sample preparation and individual structure-determination techniques, we here apply a multidisciplinary divide-and-conquer approach in which we combine data acquired with different techniques on overlapping domain variants of the hPRLR with molecular modelling. In the centre of this endeavour lies the determination of the hPRLR-TMD structure, which we here solve in 1,2-dihexanoyl-sn-glycero-phosphocholine (DHPC) micelles using nuclear magnetic resonance (NMR) spectroscopy. Combining this with previously published and new biophysical data of the soluble domains, we generate a structural model of the monomeric hPRLR, suggested to represent up to 70% of the PRLRs in the cell membrane^15^. This hPRLR structure provides the first full molecular architecture of a class I cytokine receptor, exemplifying more than 40 different receptor chains, and reveals that the extracellular domain is merely the tip of a molecular iceberg.

## Results

### Structure of hPRLR-TMD in micelles

As the missing piece in the structural description of the hPRLR, we determined the structure of the TMD in a membrane-mimicking environment using solution state NMR spectroscopy. To ensure overlap with previously determined structures, we used a hPRLR-TMD construct with a five-residue overlap between hPRLR-D2 (ref. 12) and hPRLR-TMD in its N-terminus (F206-D210), and a five-residue overlap with hPRLR-ICD^10^ in its C-terminus (G236-V240). The resulting 37-residue hPRLR-TMD harboured residues F206-V240, as well as an N-terminal G-S sequence.

**Figure 1:**
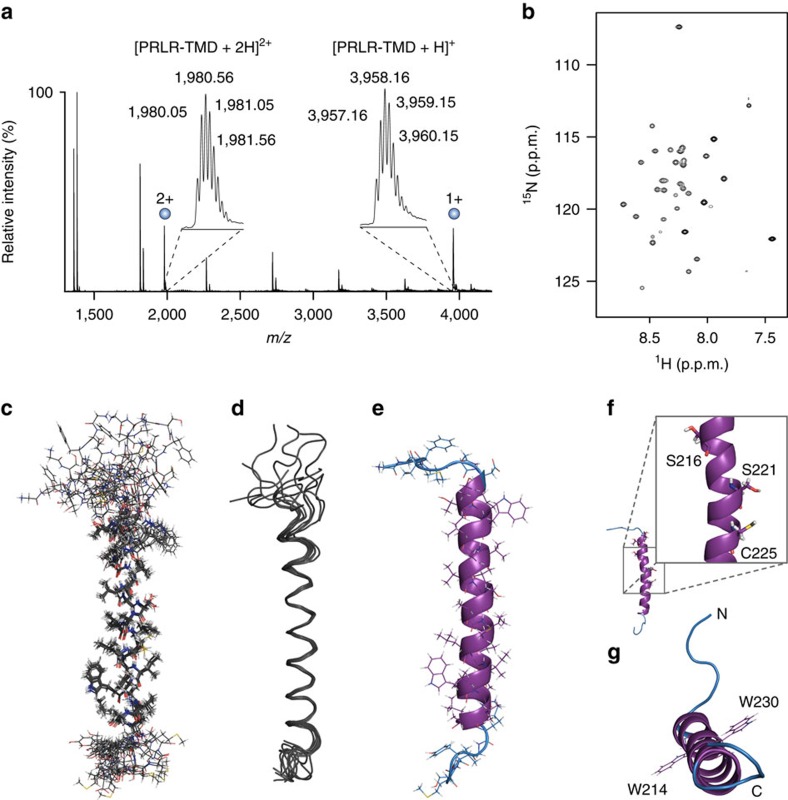
Structure of hPRLR-TMD in DHPC micelles. (**a**) Native mass spectrum of hPRLR-TMD in 700 × molar excess of DHPC. The peaks at 1,980.56 *m*/*z* (+2) and 3,958.16 *m*/*z* (+1) are consistent with one hPRLR-TMD monomer (3,959.78 Da), while no peaks representing higher oligomers of hPRLR-TMD were observed. (**b**) ^1^H-^15^N-HSQC spectrum of 1 mM hPRLR-TMD in 700 mM DHPC, 50 mM NaCl, 20 mM Na_2_HPO_4_/NaH_2_PO_4_, pH 7.2, 37 °C. Superimposition of the 10 lowest energy hPRLR-TMD structures in (**c**) stick and (**d**) ribbon representations. (**e**) Lowest energy structure of hPRLR-TMD in DHPC micelles with backbone atoms in cartoon representation. The DHPC embedded region is shown in purple. (**f**) Cartoon representation of hPRLR-TMD with polar side chains shown as sticks. (**g**) Top view of hPRLR-TMD highlighting the two symmetrically placed tryptophans.

hPRLR-TMD was expressed and fast-track purified to >95% purity (Supplementary Fig. 1) using a novel method^27^ and screened for suitable detergents and temperatures for the structural studies (Supplementary Fig. 2). A high concentration of DHPC (TMD:DHPC 1:700) at 37 °C provided narrow linewidths and the expected number of peaks and hence was selected for the structural studies. An SDS-PAGE of hPRLR-TMD (Supplementary Fig. 1) revealed a clear monomer band below 14 kDa, with a faint dimer band just above 14 kDa. The deviation from the average molecular weight of hPRLR-TMD (3,959.78 Da) can be explained by the commonly encountered gel-shifting phenomenon observed for membrane proteins^28^. The oligomeric state of hPRLR-TMD reconstituted in DHPC (1:700) was further evaluated by native mass spectrometry, which is sensitive to oligomerization of membrane proteins^29^ (Fig. 1a). The peaks identified at 1,980.56 *m*/*z* in the +2 charge state and at 3,958.16 *m*/*z* in the +1 charge state are consistent with the average molecular weight of one monomer (3,959.78 Da). No peaks representing higher oligomers of hPRLR-TMD were observed. Thus, hPRLR-TMD appeared monomeric under these conditions.

We proceeded to assign the chemical shifts of hPRLR-TMD in DHPC micelles by standard heteronuclear NMR methods. Manual assignments resulted in 99.0% completeness for backbone ^15^N, ^13^C and ^1^H resonances, and 90.3% for side chain proton resonances. The secondary structure was evaluated by secondary chemical shift (SCS) values of C^α^, C^β^, C′ and H^α^ calculated from published random coil values^30^, and with the motif identification from chemical shifts (MICS) programme^31^ using C^α^, C^β^, C′, N^H^, H^N^ and H^α^ chemical shifts. Together these analyses suggested α-helical conformation for residues D210-A233 (Supplementary Figs 3a and 4a), additionally supported by diagnostic α-helical nuclear Overhauser effects (NOEs) (Supplementary Fig. 3a). The SCSs further suggested that residues L234–G236 continued in a transient α-helical turn (Supplementary Figs 3a and 4a). The SCSs and NOE patterns of residues F206–N209 and Y237–V240 did not support highly populated secondary structures for these residues. Analysis of possible N- and C-terminal α-helical capping (N-cap and C-cap) motifs using MICS (Supplementary Fig. 4c,d) suggested N209 or D210 (0.114 and 0.118, respectively) to potentially form transient N-cap structures.

**Figure 2:**
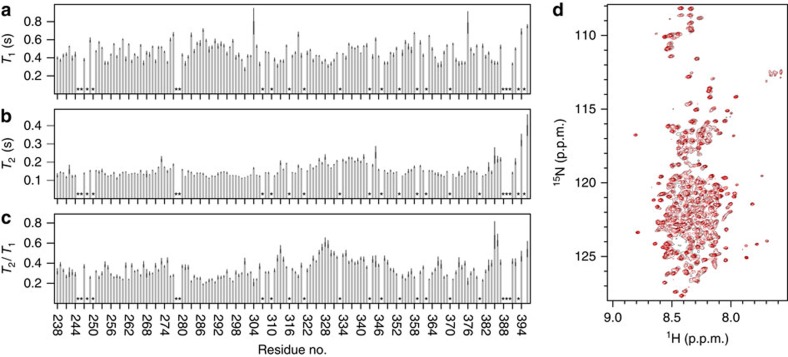
Intrinsic structural properties of hPRLR-ICD. (**a**) *T*_1_- and (**b**) *T*_2_-relaxation times for hPRLR-ICD_G236–Q396_ backbone amides plotted against residue number. (**c**) *T*_2_/*T*_1_ for hPRLR-ICD_G236–Q396_ backbone amides plotted against residue number. The vertical black lines represent the estimated error of each fit. ‘*' indicates residues for which the relaxation time was not determined. (**d**) Concentration dependence of the spectral properties of hPRLR-ICD_G236–H598_. Overlay of ^1^H-^15^N-HSQC spectra of 20 μM (red) and 400 μM (black) hPRLR-ICD_G236–H598_. The spectra were recorded under identical conditions, except that the low-concentration sample was recorded with 152 transients, while the high concentration was recorded with 16. No changes in chemical shifts were detected as a consequence of the 20 times dilution, highlighting the monomeric properties of hPRLR-ICD_G236–H598_.

The above results were supported by ^15^N-*T*_2_-relaxation times measured on hPRLR-TMD in DHPC micelles (Supplementary Fig. 3c). Little variation was observed in backbone dynamics for residues T212–L234 with ^15^N-*T*_2_-relaxation times just below 50 ms. Towards the N- and C-terminus, the relaxation times gradually increased, suggesting faster dynamics and correlating well with the lack of secondary structure in these regions. Similar relaxation measurements conducted on hPRLR-ICD_G236–Q396_ (including residues S238–V240 of hPRLR-TMD) (Fig. 2a–c) supported these findings and further showed fast backbone dynamics of the ICD.

The structure of hPRLR-TMD in DHPC micelles was determined using 754 NMR-derived restraints (Table 1), including short- and medium-range NOE-, dihedral angle- and hydrogen bonding restraints (Supplementary Fig. 3a,b). Dihedral angle restraints were estimated by TALOS^32^ and ϕ-angles further refined from ^3^J(H^N^–H^α^) coupling constants^33^ (Supplementary Fig. 3b). We used the presence of small coupling constants and characteristic α-helical NOE patterns as basis for including hydrogen bonds between H_*i*_(N) and (C)O_*i*-4_ as restraints (Supplementary Fig. 3b, grey circles). A final set of 200 structures was calculated with Aria/crystallography & NMR system (CNS),^34^,^35^ and the 10 structures with the lowest energy conformations and without significant violations were selected to represent the structure of the monomeric hPRLR-TMD (Fig. 1c,d and Table 1). The lowest energy structure formed a single α-helix from residues D210–A233 (Fig. 1e) with a length of 36 Å and two symmetrically placed tryptophans pointing in opposite directions on each side of the α-helix (Fig. 1g). A slight bend around S221 was observed, with a bending angle of 6°. The α-helix contained mainly hydrophobic residues, but harboured a few residues in the N-terminal part with side chains capable of forming hydrogen bonds: S216, S221 and C225 (Fig. 1f). S221 and C225 were located on the same side of the helix, while S216 was located half a turn away (Fig. 1f).

### Envelope structure of the unliganded hPRLR-ECD

**Figure 3:**
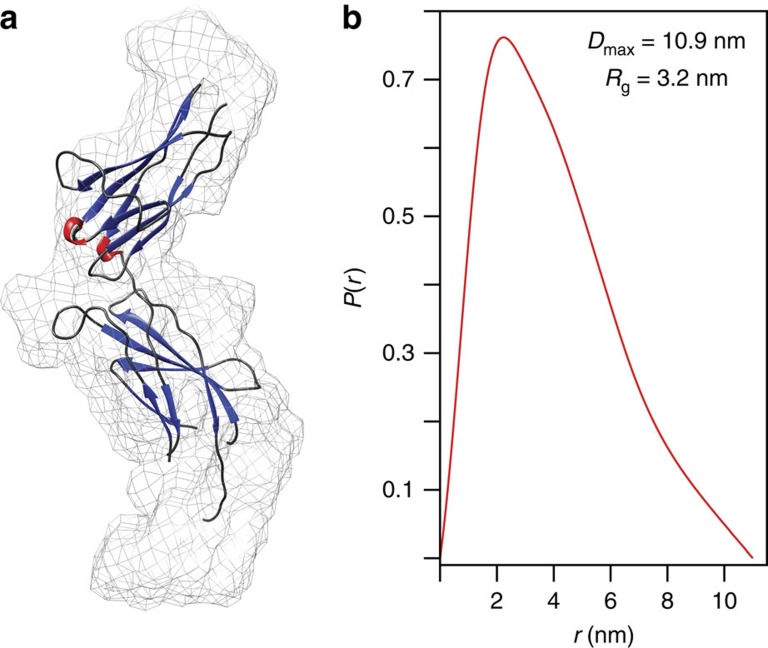
Monomeric, unliganded hPRLR-ECD envelope generated from SAXS data. (**a**) Superimposition of the SAXS ECD envelope (mesh) and a crystal structure of the liganded hPRLR-ECD (PDB entry 3D48, β-sheets are blue, α-helices are red, loops are grey) generated with UCSF CHIMERA^66^. (**b**) Pair distance distribution function of the SAXS data collected on the unliganded, monomeric ECD.

**Figure 4:**
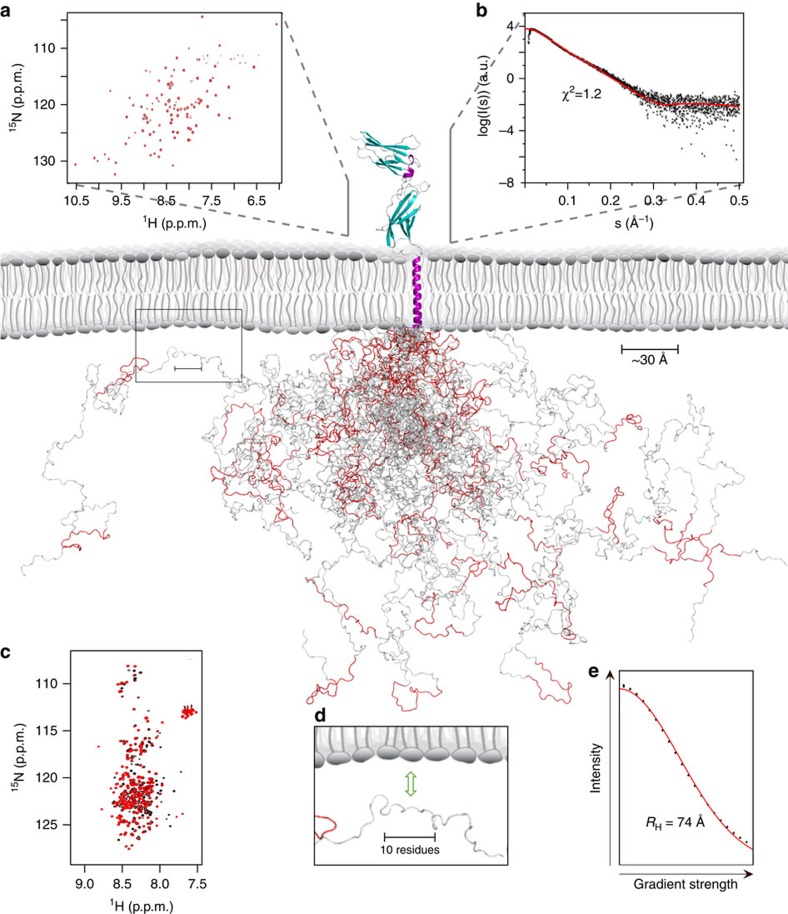
Structural model of the full hPRLR. The ICD is for clarity represented by 25 models out of the 1,000-member FM ensemble. α-helices are purple, β-sheets cyan and transient α-helices are red. (**a**) ^1^H-^15^N-HSQC spectrum of hPRLR-D2 in the presence (red) and absence (black) of POPC SUVs. (**b**) SAXS-scattering curve of monomeric hPRLR-ECD (black) fitted with the coordinates of the liganded hPRLR-ECD (PDB entry 3D48) using CRYSOL (red). (**c**) ^1^H-^15^N-HSQC spectrum of hPRLR-ICD in the presence (red) and absence (black) of POPC/POPS SUVs. (**d**) Zoom in on one ICD model. The ICD has an affinity for the inner membrane leaflet^10^ indicating that at certain times, dependent on for example the phosphorylation status of the receptor, much of the ICD will be located mainly at the membrane interface, in close vicinity to for example membrane anchored kinases. (**e**) Determination of *R*_H_ of hPRLR-ICD by pulse-field gradient NMR. The NMR signal decay of hPRLR-ICD (black) is shown as a function of field strength, together with the corresponding fit (red).

To generate a full structural model of the monomeric hPRLR, we needed a representation of the unliganded ECD. However, all known structures of the PRLR-ECD are crystal structures solved in complex with PRL, placental lactogen or PRL-based antagonists^19^,^20^,^36^,^37^,^38^. We previously attempted to solve the structure of the unliganded hPRLR-ECD in solution, but found it incompatible with acquisition of high-quality NMR data^12^. Instead, the structure of hPRLR-ECD-D2 was solved, revealing that the overall conformation of the unliganded ECD-D2 was similar to that of the liganded state, but with significant local differences in strand length and in the WS-motif^12^. Thus, to obtain structural information on the full ECD in the unliganded state, we measured solution small-angle X-ray scattering (SAXS) profiles of the unliganded hPRLR-ECD, shown to be monomeric by size-exclusion chromatography^22^ (Figs 3b and 4b). The resulting SAXS curve was fitted to the coordinates of a liganded hPRLR-ECD structure^37^ using CRYSOL^39^. The theoretical scattering curve of this ECD model fits in detail with the experimentally determined envelope (χ^2^=1.2) and a pseudo-atom model was constructed using DAMMIN (Fig. 3a,b). The angle between D1 and D2 in the ECD model was, within the resolution measurable by SAXS analysis, similar to both the liganded and unliganded GHR and EPOR structures (PDB entries 2AEW, 1A22, 1ERN and 4Y5Y). Together these findings suggested that no substantial structural rearrangement occurs upon ligand binding to hPRLR-ECD, and provided a model of the unliganded hPRLR-ECD.

### Ensemble description of the hPRLR-ICD

We recently showed the long-form hPRLR-ICD to be intrinsically disordered with five transiently populated α-helices^10^. Here, by NMR, we further investigated the intrinsic propensity of the ICD to homodimerize and found no evidence to support dimerization of the unmodified ICD chain (Fig. 2d). Thus, we used Flexible Meccano^40^ (FM) to generate a model of the hPRLR-ICD_G236–H598_ as an ensemble of monomeric, rapidly inter-converting conformers. For validation purposes, NMR diffusion experiments were applied to measure the hydrodynamic radius (*R*_H_) of hPRLR-ICD to 74 Å±1 Å (Fig. 4e). The calculated average *R*_H_ of the FM ensemble was 58±6 Å, suggesting it to be slightly more compact than proposed by the experimentally determined *R*_H_ (discussed below).

### Membrane embedment and interactions

Although the TMD is presumed to be the only truly membrane-embedded part of the hPRLR, the ECD and the ICD are tethered by the TMD to the outer or inner leaflets of the lipid bilayer, respectively. Potential interactions between the hPRLR and the membrane bilayer should therefore not be ignored. We recently established that hPRLR-ICD interacts with lipids characteristic of the inner membrane leaflet^10^, but it is unknown if—and to what extent—the ECD interacts with the membrane as suggested from theoretical considerations^41^.

Using an analogous approach as for hPRLR-ICD^10^, putative interactions between hPRLR-D2 and 1-palmitoyl-2-oleoyl-sn-glycero-3-phosphocholine (POPC), the most abundant lipid of the mammalian outer membrane^42^, were probed using NMR by titrating small-unilamellar vesicles (SUVs) into a solution of ^15^N-hPRLR-ECD-D2. However, in contrast to the ICD, we observed no effect on the chemical shifts or peak intensities on addition of 16 × molar excess of POPC (Fig. 4a), suggesting no significant affinity between the ECD-D2 and the major lipid constituent of the membrane bilayer.

Embedment of hPRLR-TMD in DHPC was examined with a series of experiments. First, the proximity and interaction between water and backbone amides were investigated using amide–water NOEs (Supplementary Fig. 5a). Not surprisingly, NOEs were readily detected between water and the amides in the flexible N- and C-terminal regions (F206-D210, K235-V240), whereas no detectable NOEs were identified from the majority of the amides in the TMD α-helix. However, we did observe NOEs between water and the amides of T212, V213, S216, V217 and S221, some of which are positioned close to the centre of the TMD. However, these NOE peaks could originate from the proximate hydroxyls of T212, S216 and S221. To further understand the TMD embedment, we therefore performed hydrogen–deuterium (H–D) exchange experiments at different levels of D_2_O following establishment of a quasi-stationary state (Supplementary Figs 5b and 6). These data supported the pattern revealed from the amide–water NOE data, suggesting some degree of water contact at the N-terminal part of the α-helix, potentially explained by this being more polar than the C-terminal (Supplementary Fig. 5c). The chemical shifts, NOEs, coupling constants and ^15^N-T_2_-relaxation times clearly established structure in the N-terminal end of the α-helix. Thus, the non-uniform exchange pattern and the water NOEs are likely not a result of extreme dynamics, but instead of the N-terminal polarity in combination with the properties of the detergent.

DHPC is, as a short-tail version of POPC, in theory only capable of assembling a ∼16 Å wide hydrocarbon bilayer^43^, compared to the 29 Å of a POPC bilayer^44^, rendering a spherical micelle-embedment model unlikely. Thus, in light of the above data, it seems likely that DHPC patches the TMD surface with a prolate ellipsoid monolayer (Supplementary Fig. 5d) as suggested also for the OmpX^43^. Despite this, the hPRLR-TMD region embedded in DHPC fits remarkably well with the region predicted to be within the native bilayer (T211–L234)^45^, possibly caused by anchoring at two charged residues; D210 and K235. Hence, even with the shortcomings of DHPC, the data collectively suggest that the membrane embedment range was well-simulated and constituted T211–L234.

In conclusion, the ICD has previously been shown to interact with membrane bilayer constituents, whereas the ECD-D2 appears intrinsically void of significant affinity for POPC. The data collectively supported that T211–L234 of the TMD were embedded in the membrane mimetics, while the extracellular F206–D210 and intracellular K235–V240 protruded at each end.

### Data integration to generate a full structural model

We combined the experimentally based ensemble of the ICD and the unliganded ECD with the structure of the TMD to generate a complete structural model of the hPRLR (Fig. 4). The overlapping region between the hPRLR-ECD and hPRLR-TMD (F206-D210) formed little regular structure, as evidenced by the SCSs from the solution structure of the ECD-D2 (ref. 12), and the coupling constants, relaxation rates and SCSs of the TMD (Supplementary Fig. 3a–c). Thus, residues P203–N209 most likely constitute a linker region without regular secondary structure between β-strand G of the ECD-D2 and the TMD α-helix, which we term juxtamembrane-linker 1 (JML1). Proceeding the TMD helix, L234–G236 continued in a transient helical turn, while we observed no regular secondary structure in the overlap region between the TMD and the ICD (G236–V240) neither in the data from this study (Supplementary Fig. 3a–c and Fig. 2a) nor from the previous characterization of the ICD^10^. We thus refer to residues Y237–C242 as juxtamembrane-linker 2 (JML2). Hence, we find that the overlap regions between the three isolated domains lacked regular secondary structure and therefore were suitable as assembly sites. We oriented the ECD perpendicular to the membrane surface, as no interactions between hPRLR-ECD-D2 and POPC were observed (Fig. 4a), while the TMD was oriented in accordance with the DHPC-embedment data presented above.

**Figure 5:**
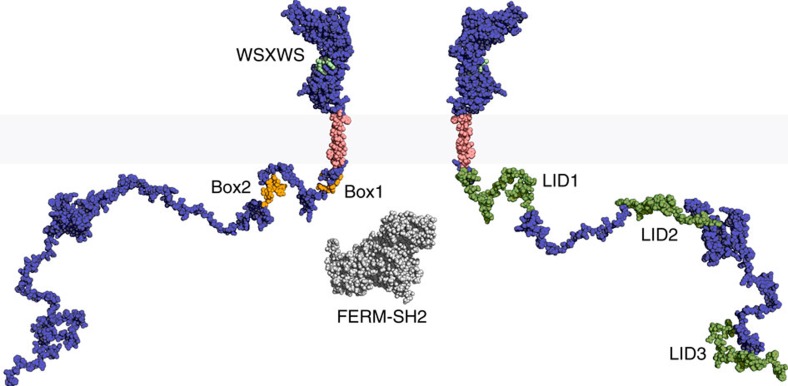
Molecular architecture of the hPRLR with important regions highlighted. The full hPRLR structural model is shown as spheres from two different angles, with the ICD represented by 1 out of the 1,000 models. The membrane-embedded part of the receptor is coloured pink, while the water-soluble domains are blue. The WSXWS motif is shown in mint green, Box1 and Box2 are orange and the three LIDs are green. For size comparison, the FERM-SH2 domains from TYK2 (PDB entry 4PO6 (ref. 56)) are shown in grey.

The resulting structure of the monomeric hPRLR provides the first view of the molecular architecture of a full class I cytokine receptor (Figs 4 and 5). Moving from N- to C-terminus, it consists of the ECD (Q1-I202), JML1 (P203-N209), TMD (D210-G236), JML2 (Y237-C242) and ICD (I243-H598). The hPRLR structure is ∼345 Å on the vertical axis from N to C terminus, of which the ECD constitute ∼20%, the TMD ∼10% and the ICD ∼70%.

## Discussion

In this work we have presented a structural model of the unliganded, monomeric hPRLR-ECD generated from SAXS data. The structure of the liganded ECD^37^ fits in detail into the unliganded ECD SAXS envelope, revealing that the relative orientation of D1 and D2 is preserved (Fig. 3a). Thus, in line with findings of time-resolved Förster resonance energy transfer (FRET) studies^46^, no substantial conformational changes appear to take place in the PRLR-ECD upon hormone binding. This observation is consistent with findings for the GHR; the crystal structure of the unliganded GHR-ECD^23^ shows only minor changes compared to the ligand bound state, pointing towards a signal transduction mechanism that rely on subunit reorientation, as suggested for the GHR^17^. However, there are also studies pointing at differences in the signal transduction mechanism between the GHR and the PRLR. For example, although the ICDs of the receptors share properties such as intrinsic disorder and conserved motifs (Box1 and Box2), they also display interesting differences in their pattern of transient structures and number and position of lipid interaction domains (LIDs)^10^. Further, in alanine-insertion studies of the GHR^23^ and the PRLR^24^, insertions in the JML2 of the GHR affected receptor activity^23^, while analogous insertions in the PRLR had no effect^24^. For the JMLs to function as hinges in the subunit reorientation mechanism they need to have some degree of rigidity. The lack of effect on insertions in the hPRLR-JML2 may be understood from the structural model presented here and previously published data^10^, showing that the hPRLR-JML2 is without rigid structure, whereas the corresponding region in the GHR showed propensity for transient α-helical structures. Thus, if JML2 in the PRLR should function as a hinge during activation, the required rigidity must be imposed by other components than the intrinsic structure alone. Overall, these findings suggest that the PRLR and the GHR may share similar activation mechanisms on the extracellular side of the receptor, but vary intracellularly in line with their different biological functions.

The structure of the monomeric hPRLR-TMD presented in this work revealed the extent of the α-helical secondary structure and a characteristic tryptophan symmetry. Tryptophans are preferably positioned towards the ends of bitropic TMD α-helices^47^, receive the greatest energy reward for partitioning into the lipid head group region^48^, and may act as interfacial anchors that regulate the helix tilt angle^49^. The placement of W214 and W230 in the second helix turn from the N- and C-termini of the TMD, respectively, fits well with a role of these in membrane anchoring. If the PRLR exerts its functions through a similar mechanism as suggested for the GHR^17^, the hPRLR-TMD should be able to switch between two different dimer conformations corresponding to the inactive and active states. With this in mind, W214 and W230 may be important not only in terms of controlling the crossing angle of the lowest energy monomer and dimer, but also in rendering a second dimerization interface, with a different crossing angle, less favourable.

Although the TMDs of class I cytokine receptors have been shown to be responsible for receptor homodimerization^16^,^17^,^18^, no classical dimerization motifs^50^ are present in the hPRLR-TMD and the monomeric form was readily obtained in this work even at high concentrations. Thus, hPRLR-TMD_206–240_ appears to have a weak inherent propensity to homodimerize. In fact, the lack of classical dimerization motifs is shared by the related hEPOR-TMD, and its structures have also only been solved in monomeric forms^51^,^52^. This weak inherent dimerization propensity suggests that other components than those of the hPRLR-TMD_206–240_/DHPC system may be important to drive TMD dimerization. Potential factors include the adjacent domains, membrane interaction partners such as specific lipid components (for example, phosphoinositides, cholesterols etc.) or accessory bound proteins (for example, JAK2). The polar residues in the core of the hPRLR-TMD α-helix (S216, S221 and C225) may be of possible relevance to homodimerization. In TMDs, such side chains are often involved in inter-monomeric hydrogen bonds, improving the stability and specificity of α-helical associations with one of the most common participants being serine^53^. Interestingly, the least common amino acid type in TM regions is cysteine^54^, suggesting that C225, placed deep within the TMD, might play a special role. A cellular mutagenesis study on the hPRLR has shown that substituting C225 with a serine decreased ligand-independent dimerization by ∼30% (ref. 18), suggesting that C225 takes part in TMD dimerization, without being vital. In hPRLR-TMD, S221 and C225 are positioned in two adjacent α-helical turns, while S216 is positioned half a turn away (Fig. 1f), suggesting that the hPRLR has the potential to form two different dimerization interfaces involving serines. These two could be interchangeable through rotation of the TMDs during receptor activation, perhaps with C225 as pivot point.

The structures of the monomeric TMDs from the related human and mouse (m) EPOR were recently solved in *n*-dodecylphosphocholine (DPC) micelles on the basis of dihedral angles obtained from TALOS using C^α^ chemical shifts only, a modest number of NOEs and backbone hydrogen bond restraints^51^,^52^. Due to a lack of restraints on the side chains of these structures, we regard them mainly as backbone structures, represented by a straight α-helix spanning residues L226-W258 for both species. Surprisingly, these α-helices continue nine residues into the JM-region without any apparent flexibility, having important implications for the mode of hormone-binding-induced signal propagation from the TMD to Box1. We note that no non-sequential NOEs for H249-L253 (hEPOR-TMD) or around H249 (mEPOR-TMD) or other data appear to support this α-helix extension^51^,^52^. In contrast, relaxation data for both EPOR-TMDs reveal increased internal dynamics from H249–P254, supporting a less-structured conformation not captured by the structures^51^,^52^. Thus, if these regions instead are interpreted as forming transient helical structures, these related structures have a similar overall topology as hPRLR-TMD.

Inherent limitations in sample preparation and individual techniques for structure determination make atomic-resolution studies of the structurally diverse hPRLR challenging. We therefore applied an approach in which data were integrated from multiple structural disciplines combining new SAXS, MS and NMR data with previously published data and molecular modelling to acquire the molecular architecture of the full hPRLR (Figs 4 and 5). This model provides the first molecular architecture of a full-length class I cytokine receptor, revealing the relative sizes of the individual domains. The hPRLR model consists of the soluble, globular ECD (Q1–I202), the unstructured JML1 (P203–N209), the membrane-embedded TMD (D210–G236), the unstructured JML2 (Y237–C242) and the intrinsically disordered ICD (I243–H598). The ICD was in a previous study shown to contain five transient α-helices (Fig. 4, red stretches) and three non-cooperative LIDs along the chain^10^, which have not been directly included in the model, but are shown in Fig. 5 (green).

The full hPRLR structure reveals that the ECD, which until recently was the only structurally characterized part of the class I cytokine receptors, merely constitutes the tip of an extensive molecular iceberg. Previously, visualizations of this receptor family have typically shown a disproportionally dominating ECD compared to the TMD and ICD, likely reflecting the overall information content available for each domain. From our structure it is now evident that the ICD is more than twice as extended in the direction of the membrane normal compared to the ECD and the TMD combined (Fig. 4). The structural flexibility and large capture radius of the ICD allows it to reach and interact with a variety of interaction partners, possibly also other receptors' ICDs. Interaction sites in disordered regions typically only constitute a few residues, so-called small linear motifs (SLiMs), and hence the ICD is geared to simultaneously interact with many kinases, phosphatases and other proteins. Box1 is an example of such a SLiM in the PRLR-ICD, shown to interact with JAK2 (ref. 13), most likely through its FERM (4.1, Ezrin, Radixin, Moesin) domain^55^. To illustrate the relative sizes of these proteins, Box1 along with the FERM-SH2 domains from TYK2 (ref. 56), a JAK2 homologue, are highlighted in Fig. 5.

The generated ensemble model of the ICD represents its unbound intrinsic structure, represented by an ensemble of 25 conformers in Fig. 4. However, in the cell the structural ensemble of the ICD may be different in several ways. First, of all the three LIDs^10^ have not been restricted to interact with the membrane in this model. Since experimental measurements of the *R*_H_ in the presence of SUVs would be dominated by signals from the SUVs, this was not attempted, but it is likely that the LID-mediated membrane contacts would result in a smaller *R*_H_ of the ICD. Thus, we regard the experimentally determined *R*_H_ obtained in the absence of SUVs as an upper limit capture radius for the ICD. Second, constitutively bound kinases have deliberately been omitted, primarily because their mutual binding sites have not been adequately described, if at all known. Last, post-translational modifications of the ICD, in particular phosphorylations, may have the potential to change the structural ensemble of the ICD^57^ and thus its compactness. Importantly, there are no indications to suggest the ICD to be folded, and the ICD does not intrinsically dimerize (Fig. 2d). Hence, other proteins or modifications such as acetylation and phosphorylations would be needed to promote ICD dimerization, in essence completely analogous to the hormone-induced dimerization of the ECD-D2 domains.

The presented structural model of the hPRLR provides important new insights on the full structure of class I cytokine receptors, and provides a framework for understanding the mechanisms related to these receptors, as for example, derived from cellular studies. However, being based on a divide-and-conquer approach, it still poses unresolved questions. First, due to the methodological restrictions on structural characterization of a PRLR variant that includes both the ECD and TMD, the exact relative orientation of these domains remains unestablished. However, in the alanine-insertion study by Liu and Brooks^24^, insertions in PRLR-JML1 did not affect receptor functionality, suggesting that the relative orientation between the ECD and the TMD is not essential. Second, it calls for reservations that the TMD structure was solved in detergent rather than a bilayer. Consequently, the angle between the TMD and the bilayer plane (helix tilt angle) remains speculative. Furthermore, although the structural envelope was obtained in the present work and the unliganded ECD-D2 structure is available^12^, a high-resolution structure of the unliganded ECD is missing. Last, the model does not include any potential interactions with the glycosaminoglycan layer, suggested to implicate the WS-motif^11^, or effects from post-translational modifications.

In conclusion, our head to toe structural model of the hPRLR provides a starting point for future refinements and may help design strategies for novel structural and functional studies. Importantly, it exemplifies the architecture of the many biologically fundamental receptors of the class 1 cytokine receptor family, and specifies a scaffold onto which a new view on cellular signalling can be built.

## Methods

### Materials

DHPC, DPC, 1-myristoyl-2-hydroxy-sn-glycero-3-phospho-(1′-rac-glycerol) (LMPG), 1-palmitoyl-2-hydroxy-sn-glycero-3-phospho-(1′-rac-glycerol) (LPPG), 1,2-dimyristoyl-sn-glycero-3-phosphocholine (DMPC), 1-palmitoyl-2-oleoyl-sn-glycero-3-phosphocholine (POPC) and 1-palmitoyl-2-oleoyl-sn-glycero-3-phospho-L-serine (sodium salt) (POPS) were purchased from Avanti Polar Lipids (Alabaster, AL). 2,2-didecylpropane-1,3-bis-β-D-maltopyranoside (MNG-3) and Amphipol A8–35 were purchased from Anatrace (Maumee, OH). 1,1,1,3,3,3-Hexafluoro-2-propanol (HFIP) and *N*-lauroylsarcosine sodium salt (sarkosyl) were purchased from Sigma-Aldrich (St Louis, MO). Sodium dodecyl sulphate (SDS) was purchased from Avantor Performance Materials (Deventer, NL). Dihexanoyl-d22-phosphatidylcholine-d9 (d_9_DHPC) was purchased from FB Reagents (Boston, MA).

### Protein expression and purification

Complementary DNA encoding the full-length long isoform of the human PRLR was amplified from IRAKp961G13133Q (RZPD German Resource Center for Genome Research).

A detailed description of the expression and purification of hPRLR-D2_M99–D210_ can be found in ref. 12, but in brief, the sequence of hPRLR-D2 (M99-D210) was sub-cloned into a pET11a vector (Novagen), transformed into competent *E. coli* BL21(DE3) cells, and expressed in ^15^N-labelled M9-media (3 g per l KH_2_PO_4_, 7.5 g l^−1^ Na_2_HPO_4_·H_2_O, 5 g l^−1^ NaCl, 1 mM MgSO_4_, 1 ml M2 trace solution, 4 g l^−1^ glucose, 1.5 g l^−1^ (^15^NH_4_)_2_SO_4_, (ISOTEC)) added 100 μg ml^−1^ ampicillin. Following collection, cells were resuspended and sonicated on ice to bring hPRLR-D2 in solution. After ammonium sulphate (AMS) precipitation (75% (w/v), stirring for 2 h at 0 °C), the precipitate was dissolved in 30 mM NH_4_HCO_3_, 100 mM NaCl and 1 mM dithiothreitol (DTT) (pH 8.0) and applied to a Sephadex G50-Fine (GE Healthcare) column (24 × 370 mm, 167 ml). hPRLR-D2 fractions were pooled, concentrated and buffer exchanged.

The sequence of hPRLR-ECD (Q1-D210) was sub-cloned into a pET11a vector (Novagen), transformed into competent *E. coli* BL21(DE3) cells, and grown in LB media (10 g per l peptone, 5 g l^−1^ yeast extract, 10 g l^−1^ NaCl, pH 7.4) at 37 °C and 180 r.p.m. Cells were induced at OD_600_=0.8 with 1 mM isopropyl β-D-1-thiogalactopyranoside for 4 h and subsequently collected by centrifugation (5,000*g*, 15 min, 4 °C). The cell pellet was resuspended in lysis buffer (40 ml l^−1^ culture 25% (w/v) sucrose, 5 mM EDTA, 1 × PBS buffer (pH 7.4), 1% (w/v) Triton X-100), sonicated on ice (2 × 3 min with 3 min rest between rounds at 50% amplitude), and the inclusion bodies (IBs) collected by centrifugation (20,000*g*, 25 min, 4 °C). This cycle was repeated a total of three times. The IBs were denatured in 6 M Urea, 10 mM β-mercaptoethanol, 50 mM Tris–HCl (pH 9.0), followed by refolding by simple dialysis into 50 mM Tris–HCl (pH 9.0), 150 mM NaCl, 10 mM cysteamin, 1 mM cystamin (3 × 4 l), protein concentration <0.1 mg ml^−1^. The refolded fraction was precipitated by addition of AMS to 75% (w/v), and left for 2 h with gentle stirring at room temperature. The precipitate was dissolved in MilliQ water and gel-filtered on a Sephadex G50-Fine column (24 × 370 mm, 167 ml) (GE Healthcare) in 0.1 M NaCl, 1 mM DTT, 30 mM NH_4_HCO_3_ (pH 9.0). Peak fractions were dialyzed against 10 mM Na_2_HPO_4_ (pH 7.4).

A detailed description of the expression and purification of hPRLR-TMD_F206–V240_ can be found in ref. 10, but in brief, the sequence of hPRLR-TMD (F206–V240) was sub-cloned into a pGEX-4T-1 vector (Amersham, GE Healthcare), containing an N-terminal glutathione S-transferase-carrier protein and a thrombin cleavage site. The plasmid DNA was transformed into competent *E. coli* BL21(DE3) cells and expressed in either unlabelled, ^15^N or ^13^C-, ^15^N-labelled M9 media (if ^13^C-labelled: ^13^C-D-Glucose, ISOTEC, if ^15^N-labelled: (^15^NH_4_)_2_SO_4_, ISOTEC) added 100 μg ml^−1^ ampicillin. The collected cells were resuspended in lysis buffer (40 ml l^−1^ culture 25% (w/v) sucrose, 5 mM EDTA, 1 × PBS buffer (pH 7.4), 1 mM PMSF), and sonicated on ice. Subsequently the IBs were collected by centrifugation (20,000*g*, 25 min, 4 °C). This cycle of resuspension, sonication and centrifugation was repeated three times. The resulting IBs were resuspended in 50 mM Tris–HCl buffer, collected by centrifugation (20,000*g*, 20 min, 4 °C), solubilized in 12 ml (per l culture) 1.5% (w/v) sarkosyl, 100 mM DTT, 20 mM Tris–HCl buffer (pH 7.4) and incubated at room temperature with gentle agitation for 3 h. Insoluble material was removed by centrifugation (12,000*g*, 20 min, 4 °C). The supernatant was dialyzed against 0.5% (w/v) sarkosyl, 10 mM NaCl, 50 mM Tris–HCl buffer (pH 7.4) to remove DTT and cleaved with thrombin to release the glutathione S-transferase carrier protein. After cleavage the solution was lyophilized, followed by resuspension in milliQ water (200 μl ml^−1^ of original solution). This solution was divided into batches of 50 μl, each of which was added to 750 μl of a 1:2 chloroform:methanol solution and mixed well. The solution was centrifuged (14,000*g*, 2 min, 4 °C), resulting in separation in three layers. The top aqueous layer was carefully removed. Subsequently, 500 μl of MeOH was added to the remaining solution, followed by thorough mixing. The mixture was incubated on ice for 20 min, followed by centrifugation (16,000*g*, 40 min, 4 °C). The supernatant containing the target protein was transferred to a glass vial, and the organic solvent evaporated under a stream of N_2_.

A detailed description of the expression and purification of hPRLR-ICD_G236–H598_ and hPRLR-ICD_G236–Q396_ may be found in ref. 27, but in brief, G236–H598 and G236–Q396 of hPRLR were sub-cloned into pET11a vectors (Novagen). The plasmids were transformed into competent *E. coli* BL21(DE3) cells and protein expressed in either unlabelled, ^15^N or ^13^C-, ^15^N-labelled M9 media added 100 μg ml^−1^ ampicillin. Cells were collected by centrifugation (20 min, 5,000*g*, 4 °C) and stored at −20 °C until thawed on ice and resuspended in 40 ml sonication buffer (20 mM Na_2_HPO_4_/NaH_2_PO_4_ pH 8, 300 mM NaCl, 0.08% (w/v) Triton X-100 and one complete EDTA-free protease inhibitor cocktail tablet (Roche Diagnostics GmbH)). The cells were sonicated on ice (4 × 30 s with 30 s rest between rounds at 100% amplitude), centrifuged (25 min, 20,000*g*, 4 °C), and the supernatants used for purification.

After sonication the supernatant containing hPRLR-ICD_G236–H598_ was heated for 5 min at 95 °C, incubated on ice for 10 min, and centrifuged to remove precipitate (10 min, 20,000*g*, 4 °C). The supernatant containing hPRLR-ICD_G236–H598_ was added 10 mM DTT, precipitated to 35% with AMS on ice, gently stirred and incubated on ice for 2 h before centrifugation (20 min, 20,000*g*, 4 °C). The pellet was resuspended in 20 ml 20 mM Tris–HCl (pH 8.0) and dialyzed against 1 l 20 mM Tris–HCl (pH 8.0) at 4 °C before applying the sample to a 5 ml HiTrap Q Sepharose Fast Flow (QFF) (Amersham Bioscience) column pre-equilibrated with 5 column volumes solution buffer (20 mM Tris–HCl (pH 8.0), 0.1 mM DTT). hPRLR-ICD_G236–H598_ was eluted over a linear gradient of 20 column volumes with elution buffer (20 mM Tris–HCl (pH 8.0), 1 M NaCl, 0.1 mM DTT).

In case of hPRLR-ICD_G236–Q396_, DNA was precipitated with a final concentration of 0.1 % (v/v) protamine sulphate added on ice, gently stirred and incubated on ice for 10 min before centrifugation (50 min, 37,000*g*, 4 °C). The supernatant was heated for 5 min at 95 °C, incubated on ice for 10 min, and centrifuged to remove the precipitate (10 min, 20,000*g*, 4 °C). The supernatant containing hPRLR-ICD_G236–Q396_ was precipitated to 35% with AMS on ice, gently stirred and incubated on ice for 2 h before centrifugation (20 min, 20,000*g*, 4 °C). The pellet was resuspended in 20 ml 20 mM Tris–HCl (pH 8.0) and dialyzed against 1 l 20 mM Tris–HCl (pH 8.0) at 4 °C before applying the sample to a 5 ml HiTrap QFF column using a protocol similar to that of hPRLR-ICD_G236–H598_.

### Screening of membrane mimetics

In case of SDS, DPC, SDS:DPC, sarkosyl, DHPC, LMPG and LPPG ∼150 nmol ^15^N-hPRLR-TMD was reconstituted in the membrane mimetics of choice in 50 mM NaCl, 20 mM Na_2_HPO_4_/NaH_2_PO_4_ (pH 7.2), followed by thorough buffer exchange in a −3 kDa cutoff spinfilter with 50 mM NaCl, 20 mM Na_2_HPO_4_/NaH_2_PO_4_ to remove residuals. When testing the two amphipols A8–35 and MNG-3, 150 nmol ^15^N-hPRLR-TMD was solubilized in 0.3% (w/v) sarkosyl mixed with either 800 nmol MNG-3 or 600 nmol A8–35. Sarkosyl was subsequently removed by thorough buffer exchange in a −3 kDa cutoff spinfilter with 50 mM NaCl, 20 mM Na_2_HPO_4_/NaH_2_PO_4_ (pH 7.2). HFIP was tested by adding 60% HFIP/40% H_2_O directly to ∼150 nmol ^15^N-hPRLR-TMD followed by thorough mixing. DHPC/DMPC-, DHPC/POPC- and DHPC/POPC/POPS (3:1) bicelles were prepared by solubilizing ^15^N-hPRLR-TMD in DHPC, followed by thorough buffer exchange in a −3 kDa cutoff spinfilter with 50 mM NaCl, 20 mM Na_2_HPO_4_/NaH_2_PO_4_. Subsequently, ((*n*_DHPC_−*n*_CMC_)/0.3) mol solubilized lipids were added to the ^15^N-hPRLR-TMD/DHPC mixture to give *q*=0.3 (long-to-short-chain lipid ratio). The TMD/DHPC/lipid mixtures were then subjected to freeze/thaw cycles until the solution cleared. The samples were prepared at 5% or 15% (w/v) lipid content. All samples were added 10% (v/v) D_2_O, 2 mM tris(2-carboxyethyl)phosphine (TCEP), 1 mM 2,2-dimethyl-2-silapentane-5-sulphonic acid (DSS), 0.05% NaN_3_ and 50 mM NaCl, 20 mM Na_2_HPO_4_/NaH_2_PO_4_ to a final volume of 370 μl followed by pH-adjustment to 7.2 before being transferred to a Shigemi NMR tube. ^1^H-^15^N-HSQC spectra of hPRLR-TMD reconstituted in the various membrane mimetics were recorded on a Varian INOVA 800-MHz (^1^H) spectrometer at temperatures between 25 and 42 °C.

### Native mass spectrometry

A sample containing 25 μM hPRLR-TMD, 700 × molar excess DHPC and 200 mM ammonium acetate buffer was prepared for native MS by extensive buffer exchange in −3 kDa spinfilter with 200 mM ammonium acetate buffer (pH 7). Samples were loaded into gold coated nano-electrospray emitters prepared in-house as previously described^58^. A range of dilutions (using milliQ water) were prepared for MS analysis, ranging from 10 to 100 μM, maintaining the 1:700 hPRLR-TMD:DHPC ratio.

### NMR spectroscopy on hPRLR-TMD

All NMR spectra were recorded on Varian INOVA 750- or 800-MHz (^1^H) spectrometers with room temperature probes or a Bruker 900-MHz (^1^H) with a 5 mM CPTCI probe. Free induction decays were transformed and visualized in NMRPipe^59^ and analyzed using the CcpNmr Analysis software^60^. Unless otherwise specified, all hPRLR-TMD samples were recorded at 37 °C and contained 10% (v/v) D_2_O, 4 mM TCEP, 2 mM DSS, 0.05% (v/v) NaN_3_, 50 mM NaCl and 20 mM Na_2_HPO_4_/NaH_2_PO_4_ (pH 7.2). Proton chemical shifts were referenced internally to DSS at 0.00 p.p.m., with heteronuclei referenced by relative gyromagnetic ratios.

For assignments of backbone nuclei, heteronuclear NMR spectra were recorded on a sample containing 0.8 mM ^13^C-,^15^N-hPRLR-TMD in 560 mM DHPC. Backbone assignments were performed manually from the analyses of ^1^H-^15^N-HSQC, HNCACB, CBCA(CO)NH and HNCO spectra acquired with non-uniform sampling^61^. The backbone chemical shifts were used in TALOS^32^ to estimate the dihedral angle restraints, and in the motif identification from chemical shifts (MICS) programme^31^ to identify possible motifs. NOE assignments were performed manually from analysis of a ^15^N-NOESY-HSQC spectrum (mixing time of 100 ms) and ^13^C-NOESY-HSQC spectra of the aliphatic region (mixing time of 150 ms) and the aromatic region (mixing time of 150 ms), acquired on a sample containing 1 mM ^13^C-,^15^N-hPRLR-TMD in 700 mM d_9_DHPC.

A 3D HNHA spectrum^33^ was recorded on 0.7 mM ^15^N-hPRLR-TMD in 490 mM DHPC and ^3^J(H^N^-H^α^) couplings constants were extracted from the relative intensity of H^α^ and H^N^ peaks using the CcpNmr Analysis software^60^. The ^3^J(H^N^-H^α^) coupling constants were utilized to estimate the backbone dihedral ϕ-angles from the Karplus relationship with coefficient values of 6.51, −1.75 and 1.60 for A, B, and C, respectively^33^. For amides with coupling constants below <5 Hz along with NOE patterns characteristic of α-helical conformation, hydrogen bond restraints were created between H_*i*_(N) and (C)O_*i*-4_.

A series of ^1^H-^15^N-HSQC spectra were recorded on a sample containing 0.7 mM ^15^N-hPRLR-TMD in 490 mM DHPC to analyze of the decay of the transverse relaxation (*T*_2_). *T*_2_ relaxation times were calculated from standard HSQC spectra recorded at 800 MHz using seven different relaxation delays between 10 and 130 ms. The relaxation decays were fitted to single exponentials and relaxation times calculated using the CcpNmr Analysis software^60^.

Hydrogen-to-deuterium (H–D) exchange experiments were performed on samples containing 0.4 mM ^15^N-hPRLR-TMD in 300 mM DHPC on the basis of the principles of Veglia *et al*.^62^ Samples were lyophilized followed by resolubilization in 10, 30, 40, 50, 70 or 90% (v/v) D_2_O. For each sample, a ^1^H-^15^N-HSQC spectrum was acquired after an incubation period of 1 h. Since the peak intensities did not change significantly between 1 and 5 h, a 1 -h incubation period was deemed sufficient for reaching a quasi-stationary state.

### Structure calculations of hPRLR-TMD

The assigned NOE peaks, dihedral angles and hydrogen bonding restraints obtained for hPRLR-TMD were applied in a standard simulated annealing protocol using Aria2 (version 2.3.2)^34^ and CNS^35^. NOE peak intensities were calibrated and converted to inter-proton distances by Aria2 during each iteration step using a distance cutoff of 6 Å. Each run consisted of eight iterations, with 20 structures calculated in each of the first seven. The seven structures with lowest global energy were used as starting structures in the subsequent iteration. The structure calculations were evaluated using the CcpNmr Analysis software^60^ and the CING suite^63^. Iteratively, assignments were checked manually, modified if needed, and structures recalculated. In the final iteration, 200 structures were generated of which the 10 lowest energy structures without significant violations (Table 1) were selected to represent the monomeric structure of hPRLR-TMD in DHPC micelles. The structures were visualized in PyMOL (DeLano Scientific). Ramachandran-plot statistics for the structure ensemble (residues 209–235) were calculated with PROCHECK^64^ and are as follows: most favored (95.2%), additionally allowed (4.4%), generously allowed (0.4%) and disallowed (0.0%).

### Lipid interaction studies of hPRLR-D2

^1^H-^15^N-HSQC spectra were acquired on samples containing 50 μM ^15^N-hPRLR-ECD-D2 in 10 mM Na_2_HPO_4_, 10 mM TCEP, 2 mM DSS and 10% D_2_O (pH 7.4) and no POPC SUVs or POPC SUVs at a final concentration of 8 mM. Chemical shift differences and intensity ratios were compared using the CcpNmr Analysis software^60^ to investigate possible interactions between hPRLR-ECD-D2 and POPC.

### SAXS and envelope generation

SAXS data on the monomeric hPRLR-ECD_Q1–D210_ were recorded at the HESYLAB synchrotron in Hamburg, beam line X33. Scattering was recorded at three different protein concentrations (1.15, 2.24 and 4.38 mg ml^−1^ in 10 mM Na_2_HPO_4_ (pH 7.4)). The three scattering curves were recorded in succession flanked by recordings of the buffer background. A high-quality scattering curve was constructed by merging the low-concentration data for low-scattering angles, intermediate concentration for intermediate-scattering angles and data from the high concentration experiment for the highest angle part of the scattering curve. The background sample consisted of the last (pure protein sample) dialysis buffer after dialysis was completed. Data were processed using the ATSAS package^65^. The theoretical scattering curve of the ECD model (on the basis of PDB entry 3D48 (ref. 37)) was fitted to the experimentally determined envelope (χ^2^=1.22) (superposition performed in CRYSOL^39^, part of the ATSAS package^65^) and the structure docked as a rigid body into the 3D density map by using the fit-in-map function from the UCSF CHIMERA^66^.

### Relaxation measurements on hPRLR-ICD_G236–Q396_

Two series of ^1^H-^15^N-HSQC spectra were recorded on ^15^N-hPRLR-ICD_G236–Q396_ to analyze the *T*_1_ and *T*_2_ relaxation times. ^1^H-^15^N-HSQC spectra were recorded at 750 MHz (^1^H) and 4°C with delay times between 10 and 1,000 ms (*T*_1_) and 10–250 ms (*T*_2_) with two triplicate measurements for each series. The relaxation decays were fitted to single exponentials and relaxation times determined using the CcpNmr Analysis software^60^.

### Oligomeric state and hydrodynamic radius of hPRLR-ICD_G236–H598_

^1^H-^15^N-HSQC spectra were recorded on ^15^N-hPRLR-ICD_G236–H598_ at 5°C in 10% (v/v) D_2_O, 8 mM TCEP, 0.5 mM DSS and 20 mM Na_2_HPO_4_/NaH_2_PO_4_ (pH 7.3), at high (400 μM) and low (20 μM) concentrations. Chemical shifts were compared using the CcpNmr Analysis software^60^ to investigate the possibility of oligomerization.

The hydrodynamic radius of hPRLR-ICD_G236–H598_ was determined by PGSLED NMR diffusion experiments using the pulse sequence of ref. 67. The experiments were performed on 800 μM ^15^N-hPRLR-ICD in 20 mM Na_2_HPO_4_/NaH_2_PO_4_, 8 mM TCEP, 0.5 mM DSS in 90% (v/v) D_2_O (pH 7). As reference, 1.5 mM α-cyclodextrin under identical buffer and experimental conditions was used. All spectra were recorded at 5 °C on a Varian Inova 750 MHz (^1^H) spectrometer. *R*_H_ of hPRLR-ICD was calculated from the relative diffusion decays of hPRLR-ICD and α-cyclodextrin^67^, which has a R_H_ of 7.52 Å (ref. 68).

### Generation of FM ensemble

An ensemble of 1,000 models of the intrinsically disordered hPRLR-ICD region (G236–H598) was generated with Flexible Meccano^40^ using default options and without any restraints. We used the HYDROPRO 10 (ref. 69) to predict the hydrodynamic properties (in particular the hydrodynamic radius *R*_H_) of each of the members of the ensemble. These values were averaged (as<*R*_H_^−1^>^−1^) to estimate the value of *R*_H_ our hPRLR-ICD ensemble. The software was used with default options, and in short describes the hydrodynamic properties of proteins by modelling the protein as a set of overlapping spheres that in turn results in a shell-model of the protein^69^.

### Structural model of the full hPRLR

The full structural model was assembled through joining the individual domains at the overlapping sequences of the structures. The overlapping regions were aligned and 1,000 model templates were constructed from the three domains by building the longest model through multiple independent cycles of refinement in Modeller 9.15 (ref. 70). As starting structures we used (1) a model of the unliganded ECD, which we built from the X-ray structure of hPRLR-ECD in complex with prolactin (PDB entry 3MZG^37^, removing the prolactin before the run) using Modeller 9.15., and which was validated by comparing calculated and experimental SAXS curves using CRYSOL^39^, (2) the NMR structure of hPRLR-TMD in micelles and (3) the FM ensemble of hPRLR-ICD, which was validated by comparing calculated and experimental *R*_H_ values as described above. Each of the 1,000 assembled models were subsequently refined by a Modeller routine through multiple cycles of conjugate gradient optimization (up to 100 steps each) optimizing the model with the variable target function method, followed by molecular dynamics with simulated annealing and a final optimization with conjugate gradients using the ‘refine.slow' option of Modeller^70^. The ECD was oriented perpendicular to the membrane surface, as no affinity for POPC lipids was observed, while the TMD was embedded in the sketch membrane in accordance with the DHPC-embedment data. Finally, we discarded all conformations of the full-length hPRLR ensemble model where the ICD folded back into regions occupied by the bilayer.

## Additional information

**Accession codes:** Resonance assignments have been deposited in the Biological Magnetic Resonance Bank under the accession code 25806. The structure of hPRLR-TMD has been deposited in the Protein Data Bank under the accession code 2N7I.

**How to cite this article:** Bugge, K. *et al*. A combined computational and structural model of the full-length human prolactin receptor. *Nat. Commun.* 7:11578 doi: 10.1038/ncomms11578 (2016).

## Supplementary Material

Supplementary Information Supplementary Figures 1-6 and Supplementary References.

## Figures and Tables

**Table 1:** NMR restraints per structure and statistics for the hPRLR-TMD structure ensemble.

	hPRLR-TMD
NMR distance and dihedral constraints	
Distance constraints	
Total NOE	677
Intra-residue	396
Inter-residue	281
Sequential (|*i*−*j*|=1)	139
Medium-range (|*i*−*j*|<4)	142
Long-range (|*i*−*j*|>5)	0
Intermolecular	0
Hydrogen bonds	13
Total dihedral angle restraints	
ϕ	47
ψ	26
	
Structure statistics*	
Violations (mean and s.d.)	
Distance constraints (Å)	0.020+/−0.002
Dihedral angle constraints (°)	1.84+/−0.001
Max. dihedral angle violation (°)	5.00
Max. distance constraint violation (Å)	0.301
Deviations from idealized geometry	
Bond lengths (Å)	0.002+/−0.000
Bond angles (°)	0.368+/−0.004
Impropers (°)	0.298+/−0.009
Average pairwise r.m.s. deviation† (Å)	
Heavy	0.40+/−0.09
Backbone	0.30+/−0.11

^*^Statistics were calculated and averaged over an ensemble of the 10 lowest energy structures out of 200 calculated structures.

^†^Pairwise r.m.s. deviation was calculated among 10 structures, and includes only the structured region of the protein.
