# Supplementary material for: A combined computational and structural model of the full-length human prolactin receptor
Source: Nat Commun. 2016 May 13;7:11578. doi: 10.1038/ncomms11578 (PMC4869255; doi:10.1038/ncomms11578)
Supplement: Supplementary Information — Supplementary Figures 1-6 and Supplementary References. [file ncomms11578-s1.pdf]

# Supplementary Information

## Supplementary Figures

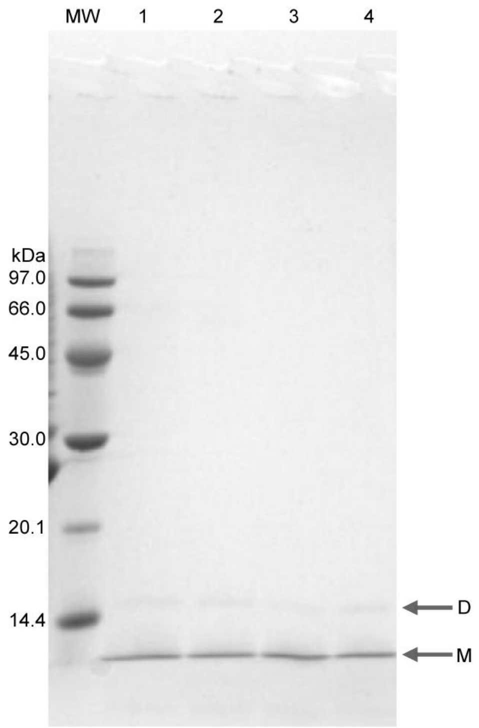


***Supplementary Figure 1. SDS-PAGE analysis of hPRLR-TMD.*** *A 15% (w/v) SDS-PAGE run under reducing conditions. Right: Molecular weight (MW) markers. Left (lane 1-4): hPRLR-TMD. The bands corresponding to monomeric and dimeric hPRLR-TMD are indicated by the arrows marked M and D, respectively.*

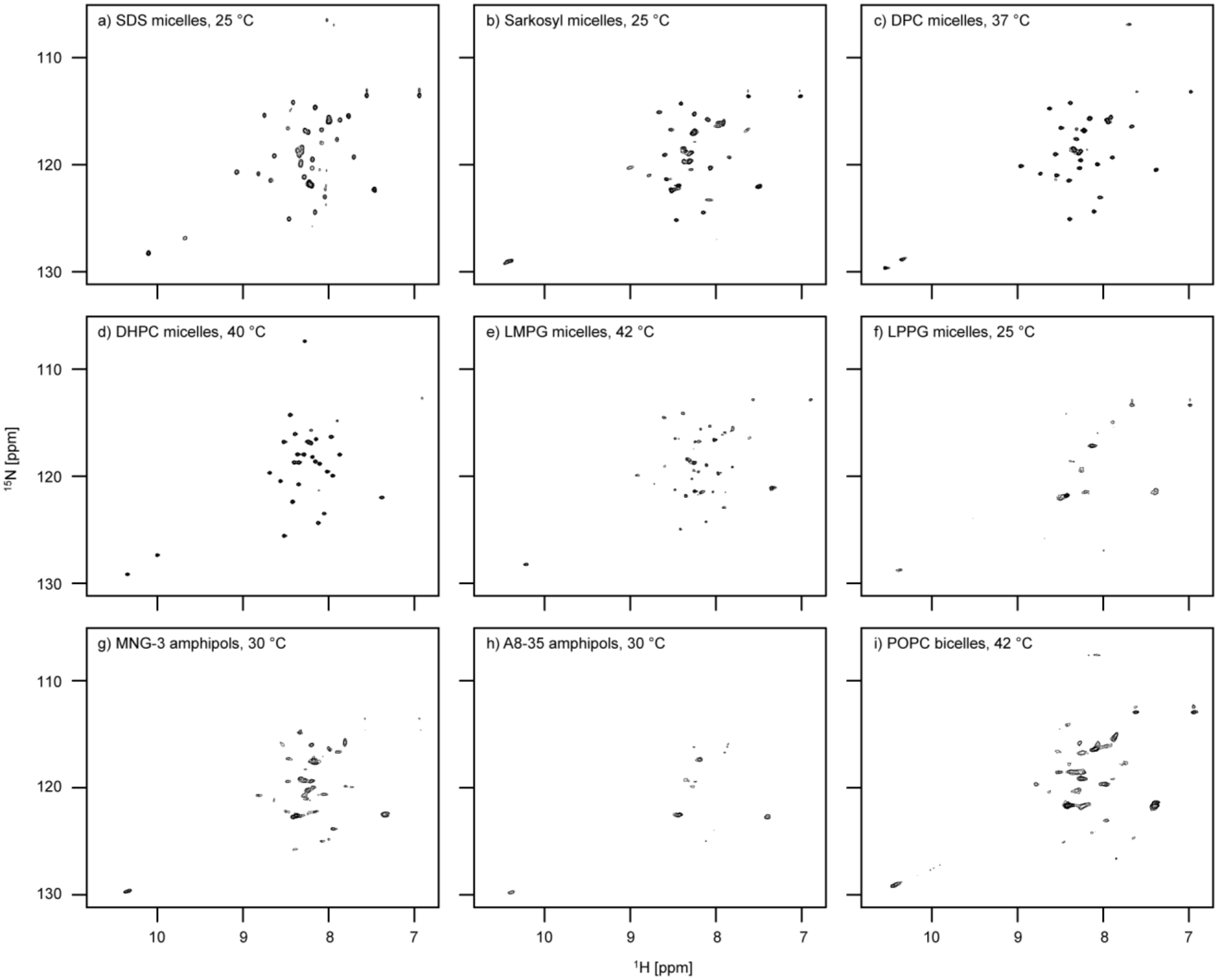


***Supplementary Figure 2. Screening of NMR-suitable membrane mimetics for hPRLR-TMD structure determination**. To obtain optimal conditions for structural studies with solution state NMR spectroscopy, hPRLR-TMD was reconstituted in various membrane mimicking solvents. A total of 13 different membrane mimetic solvents were screened: HFIP, SDS micelles, 1:2 SDS:DPC micelles, DPC micelles, sarkosyl micelles, DHPC micelles, LMPG micelles, LPPG micelles, MNG-3 amphipols, A8-35 amphipols, DHPC/POPC bicelles (q=0.3), DHPC/POPC:POPS (3:1) bicelles (q=0.3), and DHPC/DMPC bicelles (q=0.3). Furthermore, DPC micelles, DHPC micelles, LMPG micelles, LPPG micelles, and the bicelles were tested at two different concentrations. The majority of the membrane mimetics readily solubilized the TMD, but especially 60% (v/v) HFIP, and to some extent LPPG, LMPG, and the amphipols, resulted in visible aggregates. The potential for high-quality NMR data of the solubilized TMDs were evaluated from $^{1}H$-$^{15}N$-HSQC spectra at varying temperatures (25°C-40°C, not all temperatures were tested for all conditions), and a protein concentration of 0.4 mM in 50 mM NaCl, 20 mM $Na_2HPO_4/NaH_2PO_4$ (pH 7.2), and 10% (v/v) $D_2O$. Here nine selected $^{1}H$-$^{15}N$-HSQC spectra are shown of 0.4 mM hPRLR-TMD reconstituted in different membrane mimetics and at various temperatures. **a)** 80 mM SDS micelles, 25°C. **b)** 0.5% (w/v) sarkosyl micelles, 25°C. **c)** 200 mM DPC micelles, 37°C. **d)** 500 mM DHPC micelles, 40°C. **e)** 200 mM LMPG micelles, 42°C. **f)** 20 mM LPPG micelles, 25°C. **g)** 2*

*mM MNG-3 amphipols, 30°C.* ***h)*** *2 mM A8-35 amphipols, 30°C.* ***i)*** *15% (w/v) DHPC/POPC bicelles (q=0.3), 42°C. Reconstitution in some membrane mimetics resulted in broad line widths and/or a significant lower peak count than expected, revealing a high degree of variability in the quality of the resulting NMR spectra. DPC micelles and DHPC micelles provided the* $^{1}H$-$^{15}N$*-HSQC spectra of highest quality both resulting in well-dispersed peaks with narrow line widths. A closer inspection revealed that 31 out of the expected 35 peaks could be identified in the* $^{1}H$-$^{15}N$*-HSQC spectrum of hPRLR-TMD in DPC micelles, while all 35 expected peaks were resolved in DHPC micelles. Temperatures above 30 °C were found to provide the highest quality data. Based on these results, a high concentration of DHPC micelles (1:700) in 50 mM NaCl, 20 mM* $Na_2HPO_4/NaH_2PO_4$ *(pH 7.2) at 37°C was selected for the structural studies of hPRLR-TMD with solution state NMR spectroscopy. Under these conditions, the sample appeared stable for at least four weeks at 37 °C.*

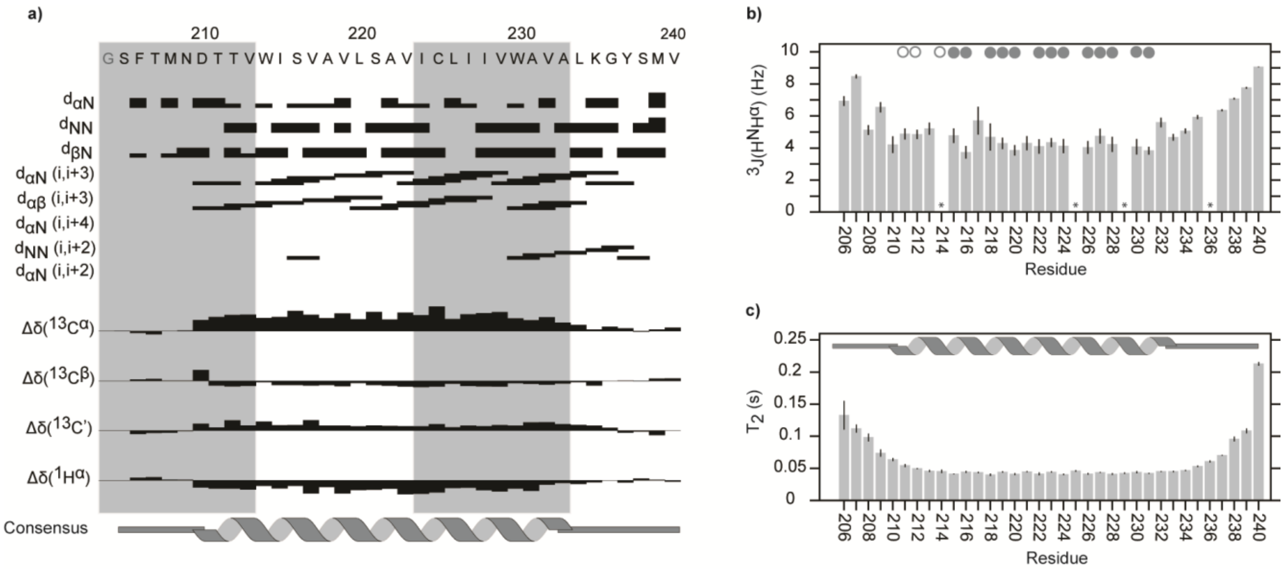


***Supplementary Figure 3. Summary of structural data collected on hPRLR-TMD in DHPC micelles. a)*** *Overview of secondary chemical shifts (SCSs) for $C^{\alpha}$, $C^{\beta}$, C', and $H^{\alpha}$ resonances and NOE connectivities within hPRLR-TMD. Positive deviations from random coil chemical shifts[1] for $C^{\alpha}$ and C' are representative of α–helical conformation, while negative deviations suggest β-sheet or extended conformations. The signs are opposite for $C^{\beta}$ and $H^{\alpha}$ SCSs. The figure has been adapted from CcpNmr Analysis software output[2].* ***b)*** *$^{3}J(H^{N}\text{-}H^{\alpha})$ coupling constants extracted from an HNHA spectrum plotted against residue number. The vertical black lines represent the estimated error of the fits for each scalar coupling constant, and "*" represents glycines or residues where the coupling constant could not be determined. The measured scalar coupling values were used to estimate the $\phi$ backbone dihedral angles using the Karplus equation[3]. Filled grey circles at the top highlight residues for which the $^{3}J(H^{N}\text{-}H^{\alpha})$ coupling constants along with characteristic NOE patterns provided the basis for inclusion of a hydrogen bond restraint in the structure calculations, while empty grey circles highlight residues only receiving the hydrogen bond restraint on their C'.* ***c)*** *$T_2$-relaxation times for hPRLR-TMD backbone amides plotted against residue number. The vertical black lines represent the estimated error of each fit.*

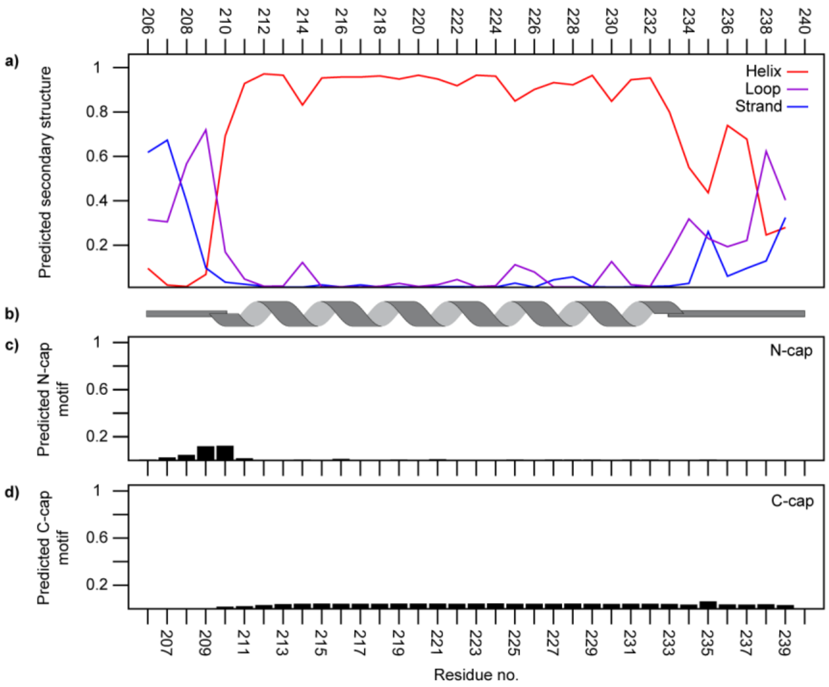


***Supplementary Figure 4. Identification of structural motifs in hPRLR-TMD using $C^{\alpha}$, $C^{\beta}$, C', $N^{H}$, $H^{N}$, and $H^{\alpha}$ chemical shifts and the motif identification from chemical shifts (MICS) program[4]. a)*** *Statistical probability calculated by MICS of finding helix (red), loop (purple), or strand (blue) elements for each residue of hPRLR-TMD.* ***b)*** *Cartoon representation of the secondary structure in the lowest energy structure of hPRLR-TMD.* ***c)*** *Statistical probability calculated by MICS of a residue in hPRLR-TMD forming an N-terminal capping motif (N-cap) plotted against residue number.* ***d)*** *Statistical probability calculated by MICS of a residue in hPRLR-TMD forming a C-terminal capping motif (C-cap) plotted against residue number.*

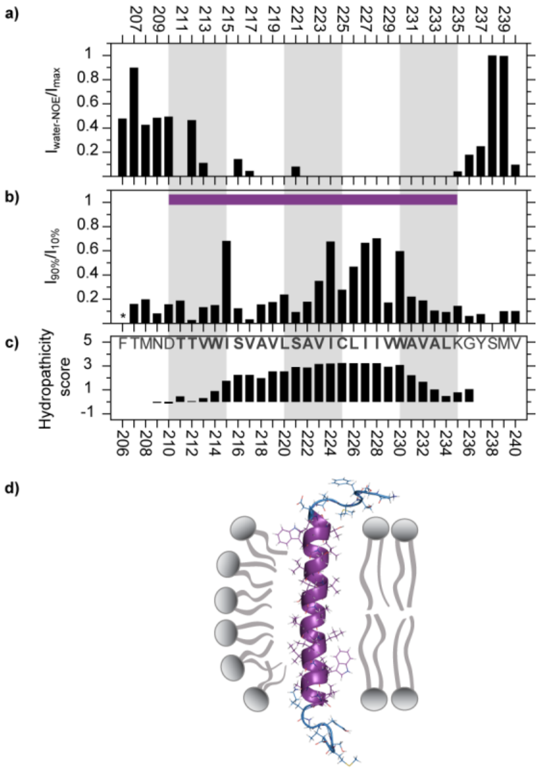


***Supplementary Figure 5. DHPC-embedment of hPRLR-TMD***. ***a)*** *Normalized intensities of NOEs between backbone amides and water plotted against residue number, extracted from a 3D* $^{15}$*N-NOESY-HSQC spectrum.* ***b)*** *Intensities of backbone amide peaks in 90%* $D_2O$ *normalized to peak intensities in 10%* $D_2O$*, plotted against residue number. The purple bar highlights the residues in helical conformation. Relatively high peak intensity indicates that the backbone amide has low exchange with water.* ***c)*** *Kyte-Doolittle hydropathy plot of hPRLR-TMD*[5]*. Positive values indicate hydrophobic stretches.* ***d)*** *Schematic model of hPRLR-TMD embedment in a DHPC micelle (left) and a lipid bilayer (right), illustrating the organizational differences of the two solvents.*

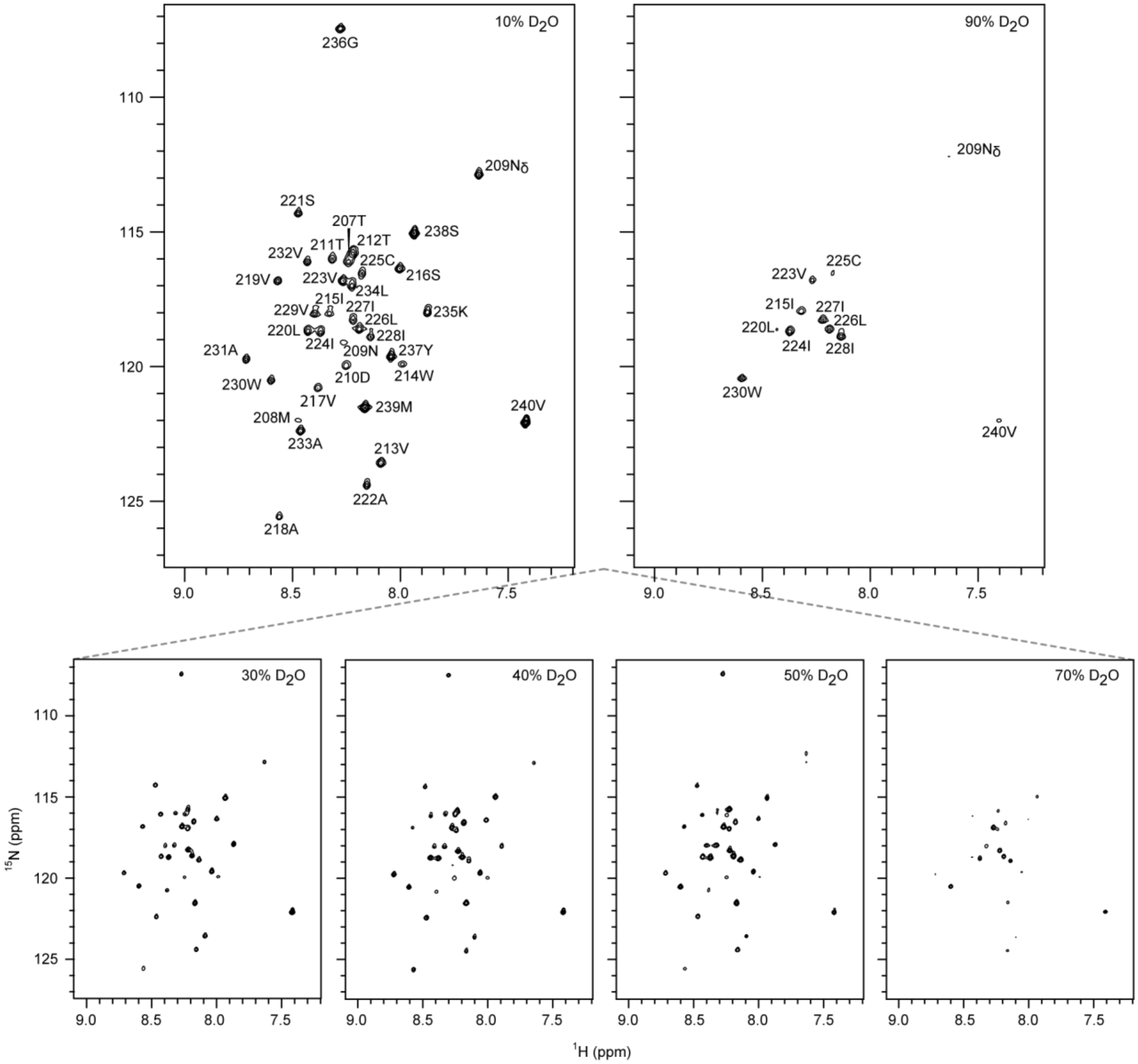


***Supplementary Figure 6. $^{1}H$-$^{15}N$-HSQC spectra from H-D exchange experiments.*** *$^{1}H$-$^{15}N$-HSQC spectra of hPRLR-TMD in DHPC micelles at different levels of $D_2O$, acquired following establishment of a quasi-stationary state.*

## Supplementary References


1. Schwarzinger, S. *et al.* Sequence-dependent correction of random coil NMR chemical shifts. *J. Am. Chem. Soc.* **123,** 2970–2978 (2001).
2. Vranken, W. F. *et al.* The CCPN data model for NMR spectroscopy: Development of a software pipeline. *Proteins Struct. Funct. Bioinforma.* **59,** 687–696 (2005).
3. Vuister, G. W. & Bax, A. Quantitative J correlation - a new approach for measuring homonuclear 3-bond J(H(N)H(alpha) coupling-constants in 15N-enriched proteins. *J. Am. Chem. Soc.* **115,** 7772–7777 (1993).
4. Shen, Y. & Bax, A. Identification of helix capping and beta-turn motifs from NMR chemical shifts. *J. Biomol. NMR* **52,** 211–232 (2012).
5. Kyte, J. & Doolittle, R. F. A simple method for displaying the hydropathic character of a protein. *J. Mol. Biol.* **157,** 105–132 (1982).
